# Correction: Effect of support on hydrogen generation over iron oxides in the chemical looping process

**DOI:** 10.1039/d4ra90038c

**Published:** 2024-04-15

**Authors:** Zhihua Gao, Fengyan Fu, Lili Niu, Min Jin, Xiaohong Wang

**Affiliations:** a Hengshui University, Department of Applied Chemistry Heping Road No. 1088 Hengshui 053000 Hebei P. R. China superman_minjin@163.com hsxygzhh@163.com; b Sichuan University Huanlu Nan No. 21 Chengdu 610041 Sichuan P. R. China

## Abstract

Correction for ‘Effect of support on hydrogen generation over iron oxides in the chemical looping process’ by Zhihua Gao *et al.*, *RSC Adv.*, 2021, **11**, 37552–37558, https://doi.org/10.1039/D1RA07210B.

The authors regret an error in the SEM data in Fig. 6, the corrected Fig. 6 is shown here.

**Fig. 6:**
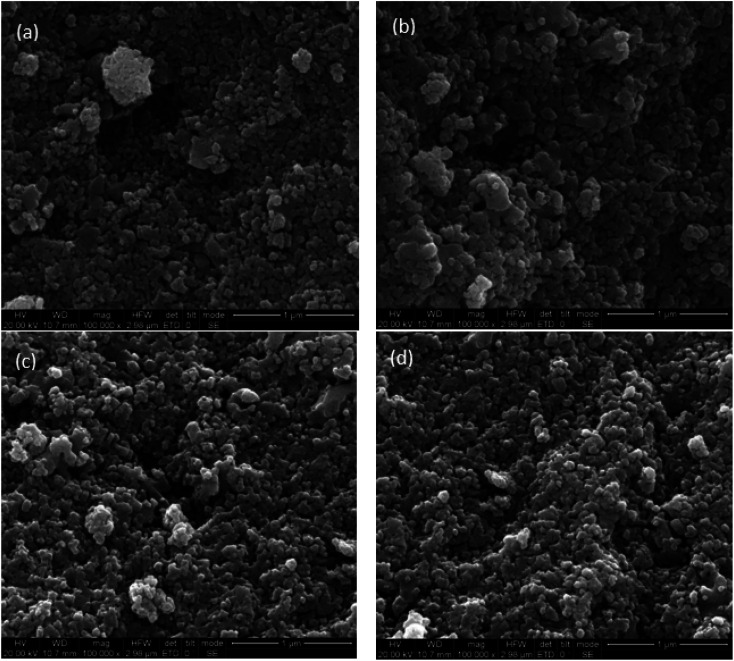
SEM of the proposed samples. (a) Fe_2_O_3_, (b) Fe_2_O_3_/MA, (c) Fe_2_O_3_/CG, and (d) Fe_2_O_3_/ZY.

An institutional investigation was carried out by Hengshui University and the reliability and integrity of the new data in Fig. 6 has been confirmed.

The Royal Society of Chemistry apologises for these errors and any consequent inconvenience to authors and readers.

## Supplementary Material

